# The mevalonate pathway regulates primitive streak formation via protein farnesylation

**DOI:** 10.1038/srep37697

**Published:** 2016-11-24

**Authors:** Yoshimi Okamoto-Uchida, Ruoxing Yu, Norio Miyamura, Norie Arima, Mari Ishigami-Yuasa, Hiroyuki Kagechika, Suguru Yoshida, Takamitsu Hosoya, Makiko Nawa, Takeshi Kasama, Yoichi Asaoka, Reiner Wimmer Alois, Ulrich Elling, Josef M. Penninger, Sachiko Nishina, Noriyuki Azuma, Hiroshi Nishina

**Affiliations:** 1 Department of Developmental and Regenerative Biology, Medical Research Institute, Tokyo Medical and Dental University (TMDU), 1-5-45 Yushima, Bunkyo-ku, Tokyo, Japan; 2 Division of Medicinal Safety Science, National Institute of Health Sciences, 1-18-1 Kamiyoga, Setagaya-ku, Tokyo, Japan; 3 Chemical Biology Screening Center, Institute of Biomaterials and Bioengineering, TMDU, Tokyo, Japan; 4 Department of Organic and Medicinal Chemistry, Institute of Biomaterials and Bioengineering, TMDU, Tokyo, Japan; 5 Department of Chemical Bioscience, Institute of Biomaterials and Bioengineering, TMDU, Tokyo, Japan; 6 Laboratory of Cytometry and Proteome Research, TMDU, Tokyo, Japan; 7 Instrumental Analysis Research Division, Research Center for Medical and Dental Sciences, TMDU, Tokyo, Japan; 8 IMBA, Institute of Molecular Biotechnology of the Austrian Academy of Sciences, Vienna, Austria; 9 Department of Ophthalmology and Laboratory for Visual Science, National Center for Child Health and Development, Tokyo, Japan

## Abstract

The primitive streak in peri-implantation embryos forms the mesoderm and endoderm and controls cell differentiation. The metabolic cues regulating primitive streak formation remain largely unknown. Here we utilised a mouse embryonic stem (ES) cell differentiation system and a library of well-characterised drugs to identify these metabolic factors. We found that statins, which inhibit the mevalonate metabolic pathway, suppressed primitive streak formation *in vitro* and *in vivo*. Using metabolomics and pharmacologic approaches we identified the downstream signalling pathway of mevalonate and revealed that primitive streak formation requires protein farnesylation but not cholesterol synthesis. A tagging-via-substrate approach revealed that nuclear lamin B1 and small G proteins were farnesylated in embryoid bodies and important for primitive streak gene expression. In conclusion, protein farnesylation driven by the mevalonate pathway is a metabolic cue essential for primitive streak formation.

In animals, the process of gastrulation involves complex movements of cells to generate the three germ layers of the early embryo that give rise to all the organs and tissues. In amniotes such as mice and primates, gastrulation involves formation of a groove of cells known as the primitive streak, which gives rise to the endoderm and mesoderm. The mouse embryonic stem (ES) cell differentiation system is a powerful *in vitro* tool for studying these fundamental developmental processes. When ES cells are cultured in suspension, they form multicellular aggregates called embryoid bodies (EBs) in a process that closely mimics early embryogenesis *in vivo*^1^. These EBs efficiently differentiate into mesodermal cardiomyocytes, but upon the addition of factors affecting primitive streak formation, the fate of the ES cells changes to ectodermal neurons^2^,^3^,^4^. Thus, this mouse EB differentiation system is a powerful tool for identifying the mechanisms of primitive streak formation. In the present study, we screened a library of 1,600 well-characterised drugs to evaluate their effect on cell fate and potentially identify new mechanisms involved in early embryogenesis (Supplementary Figure 1a). We identified 15 drugs, including atorvastatin (ATV) and lovastatin, that caused a visible change in ES cell fate from cardiomyocytes to neurons, suggesting a role in primitive streak formation (Supplementary Table 1).

Statins are used extensively in the clinic as cholesterol-lowering drugs. They work by inhibiting the mevalonate (or HMG-CoA reductase [HMGCR]) pathway, which amongst other things, leads to the biosynthesis of steroids such as cholesterol^5^,^6^. This pathway has not previously been linked with primitive streak formation, so we chose to study it in more detail. To analyse the effects of statins on EB differentiation, we performed immunostaining and quantitative real-time PCR. ATV treatment during culture days 1–6 almost completely inhibited EB differentiation into contractile cardiomyocytes and induced the formation of β-tubulin III-positive neurites by day 12 (Fig. 1a and b, Supplementary Figure 1b). Real-time PCR confirmed that the cardiomyocyte marker *Mhy7* was induced during days 8–12 in control EBs but not in ATV-treated EBs (Fig. 1b). Conversely, ATV treatment induced the expression of the neuronal markers *Map*2, *Sox1* and *Otx2* (Fig. 1b and Supplementary Figure 1c).

Next, we determined the timepoint(s) at which ATV treatment inhibited cardiomyocyte differentiation by determining the relative decrease in the number of ‘beating’ foci in an EB culture, which reflects cardiomyocyte differentiation. ATV treatment during days 1–2 or 5–6 did not decrease the beating ratio, but treatment between days 3–4 markedly reduced the number of beating foci (Fig. 1c). Western blotting (WB) and real-time PCR confirmed that ATV treatment on days 3–4 repressed the expression of the cardiomyocyte markers sarcomeric α-actinin and *Mhy7* and also induced the expression of the neuronal markers β-tubulin III, synaptophysin, neurofilament 68 (NF68) and *Map2* (Fig. 1d, Supplementary Figure 1d). ATV treatment during days 1–2 increased the levels of sarcomeric α-actinin, which might be due to the enhanced level of *HMGCR* (Supplementary Figure 1e). As an inhibitor of the mevalonate pathway, ATV blocks mevalonic acid (MVA) production. Notably, the effects of ATV were prevented by co-treatment with MVA (Fig. 1e, Supplementary Figure 1f). Therefore, the effects of ATV on EB differentiation were caused by inhibition of the HMGCR pathway.

*In vivo* primitive streak formation starts at embryonic day (E) 6 in the mouse. To assess the effect of statin on mouse embryos in the primitive streak stage *in vivo*, ATV was injected into the uteri of E5.5 pregnant mice, and the survival ratio of embryos was calculated at E10.5 (Fig. 1f and g). ATV-treated embryos had a lower survival ratio compared with DMSO-treated controls. Importantly, MVA addition partially ameliorated ATV’s effect. To investigate whether the effects of statin were conserved in other species, we treated one-cell stage zebrafish embryos with ATV and evaluated cardiomyogenesis and neurogenesis. *In situ* hybridisation of whole ATV-treated zebrafish embryos revealed that ATV disrupted heart morphogenesis in a dose-dependent manner, causing either cardia bifida or the complete absence of a heart (Fig. 1h). Real-time PCR confirmed that ATV suppressed the cardiomyocyte marker *cMhc2* in zebrafish embryos, whereas the neuroectodermal marker nestin was markedly increased (Fig. 1i and j). These data indicate that the role of the mevalonate pathway in early embryogenesis is conserved between mouse and zebrafish.

To gain initial insight into the mechanism by which statins affect early embryogenesis, we performed microarray analysis of EBs during days 3–4, which is the most sensitive two day period for ATV when compared to days 1–2 and days 5–6. ATV reduced the expression of 417 genes by 50% in EBs on days 3 and 4 (Fig. 2a). Ontology analysis revealed that many of these genes are involved in early embryogenesis, particularly gastrulation (GO: 0007369, *P* = 5.26 × 10^−31^) (Supplementary Table 2). Consistent with a role specifically in primitive streak formation, ATV markedly suppressed primitive streak and mesodermal markers (*Lhx1, Wnt3, Wnt8, GSC, Fgf10* and *Fgf8*) as well as endodermal markers (*Sox17* and *Gata6*) on day 4, which was reversed by MVA addition (Supplementary Figure 2a). In cultured mouse EBs, a primitive streak-like domain initiates a gastrulation process driven by many genes (Supplementary Figure 2b)^1^,^7^. We used real-time PCR and *in situ* hybridisation to examine the expression of the primitive streak marker Brachyury T (*T*) and the early ectodermal marker *Sox2* in mouse EBs treated with ATV. As expected, *T* was expressed at high levels in a localised region in control EBs, however levels were significantly reduced by ATV treatment (Fig. 2b,c). This effect was partially rescued by MVA (Fig. 2c and Supplementary Figure 2c). In contrast, *Sox2* levels increased in ATV-treated EBs compared with controls, and this was again reversed by MVA addition. Together, these results indicate that the inhibition of the mevalonate pathway by ATV blocks primitive streak formation. Instead, ATV-treated epiblast cells differentiate into ectoderm that gives rise to neurons (Supplementary Figure 2d).

The mevalonate pathway is important for the biosynthesis of terpenes and steroids such as cholesterol^5^,^6^. Therefore, we performed a metabolomic analysis to determine the effect of ATV on intracellular metabolite composition during primitive streak formation in EBs. Control and ATV-treated EBs were collected on days 4 and 5, and 147 metabolites were identified followed by unsupervised clustering and heat map visualisation (Fig. 3a). ATV-treated EBs had a strikingly different metabolomic profile compared to control EBs; ATV specifically downregulated 46 metabolites and upregulated 37 metabolites on days 4 and 5. We performed principal component analysis to graphically visualise the relationship between ATV-treated and vehicle-treated EB populations and days of differentiation (Fig. 3b). The first principal component distinguished ATV-treated EBs from controls, while the second principal component characterised the differences in differentiation between days 4 and 5. Sphingomyelins (d18:1/16:0), sphingosine and sphingomyelins (d18:1/18:0) were the main contributors to the first principal component (Fig. 3c, Supplementary Table 3). Interestingly, the free fatty acids (FAs) palmitic acid (16:0), stearic acid (18:0) and oleic acid (18:1) were increased following ATV treatment, whereas intermediates of FA biosynthesis from acetyl-CoA, namely palmitoylcarnitine (16:0), acylcarnitine (18:0) and acylcarnitine (18:1) were decreased (Fig. 3d). Statins also enhanced the expression of the FA synthase (FAS) gene (Supplementary Table 4), together suggesting that statins enhance lipogenesis but suppress the catabolic β-oxidation of FAs. It has been reported that FAS-dependent *de novo* synthesis of FA is enhanced in active neural stem and progenitor cells (NSPCs) and is crucial for NSPC proliferation^8^,^9^. These observations support the idea that a statin-dependent increase in free FA and FAS activity would promote neurogenesis. On the other hand, statins markedly decreased amino acids and dipeptides such as threonine, arginine and Gly-Asp (Supplementary Table 3), which might contribute to the inhibition of the primitive streak formation. Surprisingly, given the major role of statins in inhibiting cholesterol production, the intracellular cholesterol level in ATV-treated EBs was actually higher than in control EBs (Fig. 3e). Cholesterol homeostasis is maintained by a balance of *de novo* synthesis and incorporation^10^. Thus, the observed elevation in intracellular cholesterol might be due to enhanced expression of the low-density lipoprotein receptor (Supplementary Table 4), which takes up extracellular cholesterol and is induced by statins^11^. Together, our results indicate that the inhibition of statin-mediated primitive streak formation is not caused by a decrease in intracellular cholesterol but is associated with other metabolic anomalies.

To identify the signalling pathways underlying statin-induced inhibition of primitive streak formation, we analysed the role of cholesterol, geranylgeranyl diphosphate and farnesyl diphosphate, which are all downstream of the mevalonate pathway (Supplementary Figure 3a). Similar to ATV treatment, a farnesyltransferase inhibitor (FTI-277) almost completely abolished cardiomyogenesis in EBs. In contrast, a squalene synthetase inhibitor (zaragozic acid), which blocks the biosynthesis of cholesterol, and a geranylgeranyltransferase inhibitor (GGTI-2133) did not suppress cardiomyogenesis (Fig. 4a, Supplementary Figure 3b and c). Consistent with the effect of ATV on primitive streak formation, real-time PCR confirmed that expression of the primitive streak genes *T* and *Lhx1* were also markedly suppressed by FTI-277 (Fig. 4b, Supplementary Figure 3d). Moreover, FTI-277 inhibited primitive streak formation and induced *Sox2* expression (ectodermal differentiation) in a dose-dependent manner (Supplementary Figure 3f). Farnesyltransferase is an enzyme transferring farnesyl diphosphate to the cysteines at the C-terminus of proteins. Importantly, farnesol rescued *T* expression in ATV-treated EBs, confirming the involvement of farnesyl diphosphate in primitive streak formation (Fig. 4c). These data indicate that the inhibition of farnesyl diphosphate inhibits primitive streak formation, similar to statin treatment.

Farnesylation is a protein modification in which a farnesyl group is covalently attached to cysteine residues within carboxyl-terminal CaaX motifs^12^. To identify the farnesylated proteins involved in EB primitive streak formation, we used a comprehensive tagging-via-substrate (TAS) approach^13^,^14^. (Supplementary Figure 4a). Several ~20 kDa farnesylated proteins were present in both the control ES cells and EBs (black arrowheads in Fig. 4d). These spots corresponded to several small G proteins, including the well-known farnesyl substrates H-, N- and K-Ras and Cdc42, all of which have crucial roles in early embryogenesis^15^,^16^,^17^. In the present study, we focused on the 42 kDa spot appearing specifically in EBs (white arrowhead in Fig. 4d and Supplementary Figure 4b).

LC-MS/MS analysis of the 42 kDa farnesylated protein spot identified it as lamin B1 (Supplementary Figure 4c). To examine the subcellular localisation of lamin B1 in mouse ES cells, we introduced Myc-tagged wild type (WT) lamin B1 or a mutant lamin B1C-S in which the cysteine residue of CaaX is changed to serine so it can no longer be farnesylated^18^. WT farnesylated lamin B1 localised to the nuclear membrane, whereas non-farnesylated mutant lamin B1 accumulated in the nucleoplasm (Fig. 4e). These data confirmed that lamin B1 is tethered to the nuclear membrane by C-terminal farnesylation as previously shown^19^. To determine the biological function of lamin B1 in primitive streak formation, we used haploid genetics to generate lamin B1-deficient (KO) and lamin B1-revertant (REV) clones (Supplementary Figure 5a and b)^20^. Lamin B1 REV ES cells are positive isogenic controls directly derived from the lamin B1 KO cell line by inducing Cre expression and thereby reactivating expression of lamin B1, which excludes any effect of genetic background. EBs were prepared from the WT, KO and REV ES cell lines (Supplementary Figure 5c and d). We used real-time PCR to examine marker genes of primitive streak formation. Actin (housekeeping gene) expression did not differ among WT, KO and REV EBs (Fig. 4f). However, expression of the primitive streak marker *T* and the mesodermal marker *Lhx1* was markedly suppressed in KO EBs compared with WT and REV EBs. These results indicate that Lamin B1 has a crucial role in the induction of primitive streak genes.

Importantly, the U.S. Food and Drug Administration has classified ATV to pregnancy category X, meaning that the risks involved in use of the drug in pregnant women clearly outweigh potential benefits. However, our data show that the mevalonate (HMGCR) pathway is essential for the formation of the primitive streak in cultured EBs and in mice (Fig. 5, Supplementary Figure 3), and suggest an embryotoxic effect of statin on mammalian embryos, which should help better inform these guidelines.

During mouse embryogenesis, primitive streak formation occurs during E6–7.5. Consistent with our *in vitro* and *in vivo* results, *Hmgcr* KO mice die by E8.5^21^ and *FTase* KO mice succumb by E7.5^22^ (Fig. 5). We also identified farnesyl transferase downstream of the mevalonate pathway, which mediates the farnesylation of various proteins. We applied TAS technology to our ES cell differentiation system and showed that various farnesylated proteins were expressed in normal ES cells and EBs (Fig. 4d, Supplementary Figure 4a). Among these, we identified ~20 kDa farnesyl spots that corresponded to several small G proteins, including Cdc42 and Ras proteins. Loss of one of these small G proteins is typically embryonic lethal. *Cdc42* KO mice die at ~E7.5^16^, and *K-Ras* KO mice die between E12.5 and birth^15^. *H-Ras*/*K-Ras* double KO mice survive until E11.5, and *H-Ras/K-Ras/N-Ras* triple KO mice died much earlier. Together, these data suggest a key role for farnesylation in multiple stages of embryogenesis.

Our genetic and biochemical approaches coupled with our *in vitro* ES cell system allowed us to discover a novel function for Lamin B1 in primitive streak formation. Our Lamin B1 REV ES cells were directly derived from the Lamin B1 KO cell line (Supplementary Figure 5), possibly explaining how we were able to detect a subtle phenotype missed in previous studies^23^,^24^,^25^. Recently, it was revealed that chromatin-Lamin B1 interactions are tightly linked to gene repression during ES cell lineage commitment^26^. Our study indicates that the genome-nuclear lamina interactions are also involved in the primitive streak formation.

## Methods

### ES cell culture and differentiation

Feeder cell-independent E14K ES cells were maintained in gelatin-coated dishes with Dulbecco’s modified Eagle’s medium (Gibco) containing 15% Bovine Calf Serum (Hyclone and Equitech-bio), 0.1% 2-mercaptoethanol (Sigma) and 1000 U/ml LIF (propagation medium), as described previously^2^,^3^,^4^. To make EBs, 3 × 10^3^ ES cells were cultured in a 25 μl hanging drop without LIF. After 2 days, the EBs were collected and transferred into a bacterial Petri dish (IWAKI). On day 6, the EBs were transferred into a gelatin-coated tissue culture dish (Corning). Areas of tissue showing a spontaneous ‘heartbeat’ were detected by microscopy during days 10–12.

### Compound screening

The drug library (FKL series, the Chemical Biology Screening Centre of Tokyo Medical and Dental University) consisting of “The US Drug Collection” (MicroSource Discovery Systems) and “The International Drug Collection” (MicroSource Discovery Systems) was used. 1,600 chemical compounds were supplied as 100 μM stock solutions in 10% DMSO^27^. EBs were cultured in low cell adhesion 96-well plates (PrimeSurface-U, Sumitomo Bakelite Co., Ltd.) for the first 6 days. During days 2–6, EBs were treated with 10 μM of each compound. On day 6, treated EBs were transferred to gelatin-coated 96-well plates and allowed to attach to the bottom. During days 10–12, cardiomyogenesis was evaluated by microscopic observation of tissue ‘beating’, and neurogenesis was assayed by detecting β-tubulin III-positive dendrites.

### Reagents and antibodies

GGTI-2133, FTI-277, and zaragozic acid were purchased from Sigma. Antibodies (Abs) used in this study were: anti-β-tubulin III (Tuj-1, Covance, MMS-435P), anti-sarcomeric α-actinin (Abcam, ab9465), anti-GAPDH (Millipore, MAB374), anti-synaptophysin (Invitrogen, 18–0130), anti-NF68 (Sigma, N5139), anti-Lamin B1 (Abcam, ab16048) and anti-MYC (Santa Cruz, sc-40). All antibodies were used in 1:1000 dilution.

### Plasmids

FLAG-MYC-Lamin B1-pcDNA3.1 and FLAG-MYC-Lamin B1 (amino acids 234–588)-pcDNA3.1 were constructed by inserting DNA fragments that were PCR-cloned from a cDNA library of mouse ES cells into a FLAG-MYC-pcDNA3.1 vector. Lamin B1 C-S mutants were constructed by site-directed mutagenesis^28^.

### Immunofluorescence staining

Immunofluorescence staining was performed as described previously with slight modifications^29^. Cells were fixed in 4% paraformaldehyde (PFA) in phosphate-buffered saline (PBS) and washed three times with PBS. After preincubation with blocking solution (1% BSA, 0.1% Triton X-100 in PBS) for 1 h, cells were incubated overnight at 4 °C with anti-β-tubulin III or anti-MYC Ab. After washing with PBS/0.1% Triton X-100, cells were incubated with a 1:1000 dilution of Alexa Fluor 488-conjugated anti-mouse Ab (Molecular Probes) plus 1 μg/ml Hoechst 33342 (Molecular Probes). Digital images were acquired using a OLYMPUS IX71 fluorescent microscope equipped with digital camera (Digital Sight, Nikon) or a Carl Zeiss confocal microscope equipped with LSM510 software. Exposure time was 5 s and 400 ms for β-tubulin III and MYC detection, respectively.

### Real-time PCR analysis

RNA isolation and RT-PCR were performed as described elsewhere^30^. Briefly, ES cells or EBs were collected and immediately suspended in TRI reagent (Molecular Research Centre). RNA was extracted following the manufacturer’s instructions. Total RNA (1–3 μg) was used to synthesise cDNA using 500 ng oligo-d(T) primers. cDNA synthesis was performed at 42 °C for 90 min using Superscript III reverse transcriptase (Invitrogen) according to the manufacturer’s instructions. Quantitative real-time (RT)-PCR reactions were performed using the Chromo4 real-time detection system (Bio-Rad). Primer sequences are listed in Supplementary Table 5. mRNA levels were normalised to *Gapdh* and *Actin* expression for mouse and zebrafish genes, respectively^31^.

### Western blotting

WB was performed as previously described^30^, with slight modifications. Proteins were extracted in RIPA buffer, fractionated by SDS/PAGE and transferred to PVDF membranes. Blocked membranes were incubated with primary Abs, washed in TBS/0.05% Tween 20 and incubated with horseradish peroxidase-conjugated secondary Abs (Jackson ImmunoResearch Laboratory). Proteins were visualised using ChemiDoc XRS (Bio-Rad). All full blots are shown in Supplementary Figure 6.

### Statement of ethical animal experimentation

All procedures were performed in accordance with a protocol approved by the Tokyo Medical and Dental University Animal Care Committee. All experiments were conducted so as to minimise pain and discomfort.

### *In utero* statin treatment in mice

Pregnant ICR mice (Nihon SLC) were reared under a normal 12 h light/dark cycle. The day of insemination was designated as embryonic day (E) 0. One mM ATV or 1 mM ATV plus 100 mM MVA diluted in PBS was injected into the left and right sides of the uteri at E5.5 (150 μl per side). Control mice were injected with 1% DMSO/PBS. At E10.5, the injected mice were sacrificed, and uteri and morphological changes in the embryos were examined under a light microscope.

### Whole-mount *in situ* hybridisation of zebrafish embryos and mouse EBs

Wild type TL strain zebrafish were maintained at 27–28 °C under a controlled 14 h light/10 h dark cycle. Embryos were obtained from natural spawnings^32^. Zebrafish whole-mount *in situ* hybridisation was performed using antisense DIG-labelled *cMhc2* riboprobes^33^,^34^. Whole-mount *in situ* hybridisation of mouse EBs was performed using the following primers: mouse *T*, 5′-TTTGAATTCC AGTTAATCAG AGTCCTTTG-3′ and 5′-TTTAAGCTTA CCAGGTGCTA TATATTGCC-3′; mouse *Sox2*, 5′-TTTGAATTCA AACCGTGATG CCGACTA-3′ and 5′-TTTAAGCTTA TCCGAATAAA CTCCTTCCTT G-3′.

### Microarray and gene ontology analysis

Total RNA was extracted from mouse ES cells using TRI reagent (Molecular Research Centre) and further purified using the RNeasy Mini Kit (Qiagen) as described previously^32^. The quality of RNA was initially assessed by electrophoresis on a 1.5% agarose gel and later by absorption spectrophotometry [Agilent Bioanalyzer 2100 (Agilent, Palo Alto, CA)]. cDNAs were synthesised using the Low Input Quick Amp labelling kit. Cy3-labelled cRNA was synthesised by *in vitro* transcription with T7 RNA polymerase. Following fragmentation, cRNA (0.6 μg) was hybridised for 17 h at 65 °C to the SurePrint G3 mouse GE 8 × 60 K microarray using a Gene Expression hybridisation kit. GeneChips were washed using the Gene Expression wash buffers and scanned using an Agilent DNA microarray scanner (G2565CA). Microarray data analysis was conducted using KeyMolnet software (Institute of Medicinal Molecular Design Inc., Tokyo, Japan).

### Metabolomic analysis

Metabolomic analyses were performed as described previously^5^. For CE-TOFMS analysis, EBs (100) were washed once with 10 ml 5% mannitol solution and then with 2 ml of the same solution. EBs were treated with 800 μl methanol for at least 30 s to inactivate enzymes. Cell extracts were incubated with 550 μl Milli-Q water containing internal standards (Solution ID: H3304-1002, Human Metabolome Technologies, Inc., Tsuruoka, Japan) for at least 30 s. Extracts were centrifuged at 2,300 × g at 4 °C for 5 min, and 800 μl of the upper aqueous layer was recovered and filtered through a Millipore 5-kDa cutoff filter by centrifugation at 9,100× g at 4 °C for 4 h to remove proteins. For LC-TOF-MS analysis, EBs were washed twice with 10 ml 5% mannitol solution and treated with 1 ml ethanol containing internal standards to inactivate enzymes.

### Analysis of farnesylated proteins

The TAS technique was performed as previously described^13^ with slight modifications. ES cells (2 × 10^6^) and EBs (200) were treated with 25 μM farnesyl azide (Molecular Probes and Thermo Scientific) plus 10 μM ATV for 24 h to metabolically label farnesylated proteins. ES cells and EBs were homogenised, and extracts containing farnesyl azide-modified proteins were incubated with 250 μM biotin-phosphine (Molecular Probes and Thermo Scientific) at room temperature for 16 h to selectively ligate azide and phosphine groups via the Staudinger-Bertozzi reaction. Unreacted probes were removed by chloroform-methanol precipitation. For modified TAS technology, 3xFLAG-phosphine prepared by Operon, Inc. was used instead of biotin-phosphine. Cell lysates (150 μg) were subjected to 2D-PAGE using an immobilised pH gradient for first-dimension isoelectric focusing (IEF) (ZOOM IPGRunner System; Invitrogen) according to the manufacturer’s instructions, with slight modifications. Biotinylated proteins and 3xFLAG-modified proteins were detected using streptavidin-HRP and anti-FLAG Ab, respectively. Mass spectrometry analysis was performed using LC-FT-MS/MS (Thermo Scientific, LTQ Orbitrap Velos).

### Preparation of Lamin B1-deficient mouse ES cells

Lamin B1-targeted ES cell clones were generated using haploid genetics as described previously^20^. Mutagenesis of haploid cells to disrupt Lamin B1 function was performed with a polyA-trap cloned into a Tol2 transposon vector modified from Mayasari NI *et al*.^35^ (Elling, Wimmer *et al*. unpublished). To retrieve Lamin B1 gene function in the same clone, the stably integrated polyA trap was flipped by transient expression of Cre-recombinase (described in Mayasari *et al*.). These ES clones (mCherry-positive and GFP-negative) were sorted by FACS. This step was performed to minimize the probability of clonal effects (Supplementary Figure 5).

### Statistical analysis

All experiments were performed in triplicate. Data are presented as means ± standard deviation. In most instances, statistical significance was examined using the Student’s t-test. Unsupervised clustering and visualising by heat map were performed using PeakStat software (ver.3.18, Human Metabolome Technologies, Inc.). Unsupervised principal component analysis was performed using SampleStat software (ver.3.14, Human Metabolome Technologies, Inc.).

## Additional Information

**How to cite this article**: Okamoto-Uchida, Y. *et al*. The mevalonate pathway regulates primitive streak formation via protein farnesylation. *Sci. Rep.* **6**, 37697; doi: 10.1038/srep37697 (2016).

**Publisher's note:** Springer Nature remains neutral with regard to jurisdictional claims in published maps and institutional affiliations.

## Supplementary Material

Supplementary Information

## Figures and Tables

**Figure 1:**
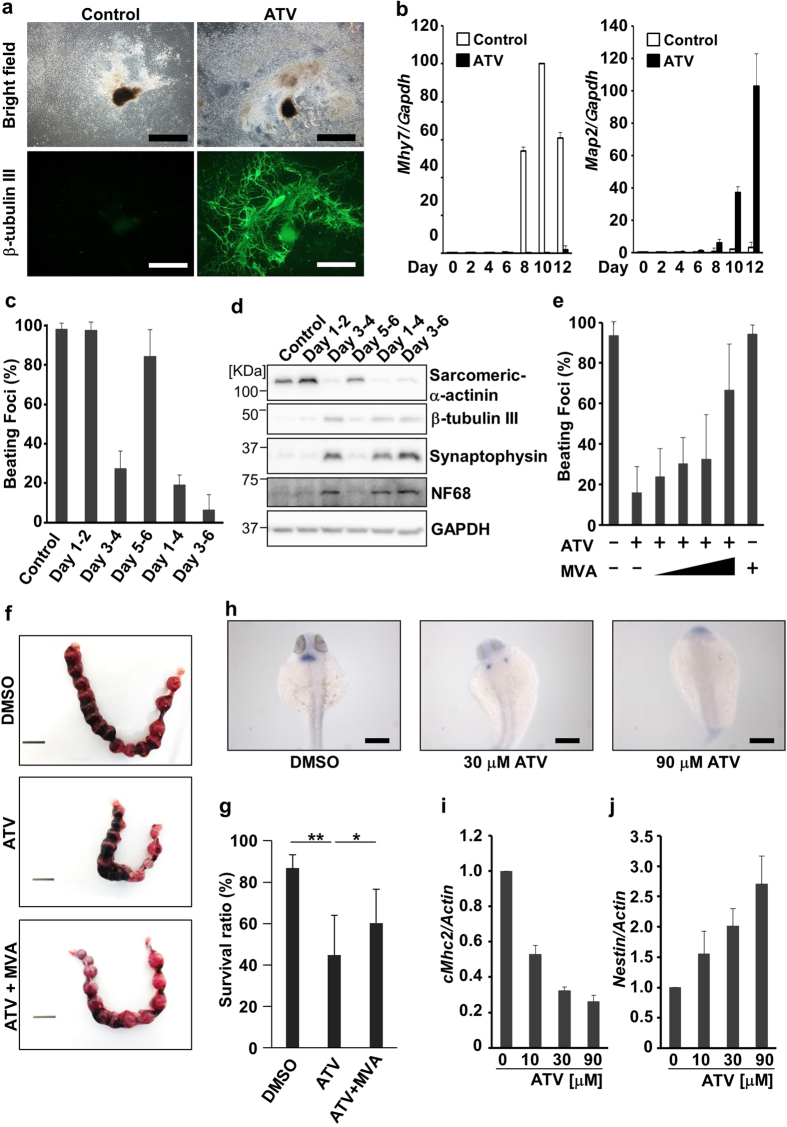
The effects of statins on cardiomyogenesis and neurogenesis. (**a**) Representative images of EBs that were untreated (control) or treated with 10 μM ATV (n > 100/group) during days 1–6. EBs were stained with anti-β-tubulin III Ab to label neurites on day 12 and visualised by microscopy. Scale bars, 1 mm. (**b**) Real-time PCR of the cardiomyocyte marker *Mhy7* and neural marker *Map2* in EBs in (**a**). mRNA levels were normalised to *Gapdh* expression. Results are means ± SD (n = 3). (**c**) Percentage of foci with a ‘heartbeat’ (indicating cardiomyocyte differentiation) in EB cultures that were treated with 10 μM ATV for the indicated periods and evaluated on day 10. Results are means ± SD (n = 3). (**d**) Western blotting of EBs in (**c**) to detect protein expression on day 10. GAPDH, loading control. Results are representative of three trials examining at least three cultures/group. (**e**) Cardiomyocyte differentiation in EBs treated with 25, 100, 250 μM or 1 mM MVA in addition to 10 μM ATV during days 3–6. Results were analysed as in (**c**). (**f**) Representative images of the uteri of mice treated with DMSO, ATV or ATV plus MVA at E5.5 and examined at E10.5. Scale bars, 10 mm. (**g**) Survival ratios for embryos of mice in (**f**). (**h**) Whole-mount *in situ* hybridisation to detect the cardiac marker *cMhc2* in 24 h post-fertilisation (hpf) zebrafish embryos that were treated with DMSO or treated with 30 or 90 μM ATV from one-cell stage. Scale bars, 200 μm. (**i,j**) Real-time PCR of *cMhc2* and *nestin* in the zebrafish embryos in (**h**) at 24 hpf. mRNA levels were normalised to actin expression. Results are means ± SD (n = 3). All experiments were carried out in triplicate. *P = 0.052, **P < 0.05.

**Figure 2:**
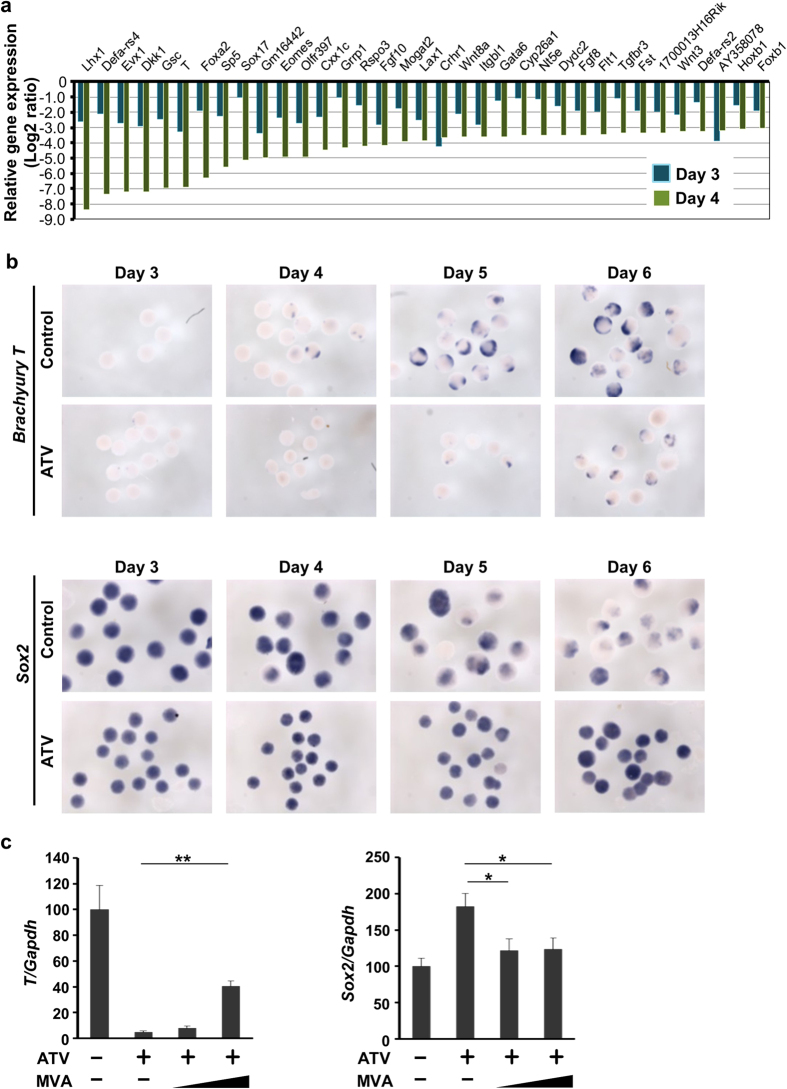
The effects of statins on primitive streak formation in mouse EBs. (**a**) Microarray results for the top 35 downregulated genes in EBs treated with ATV during days 1–4. Data were compared with control EBs and analysed on days 3 and 4. Gene expression levels in ATV-treated EBs are expressed as Log2 (fold change) values relative to control EBs. (**b**) *In situ* hybridisation to detect *T* and *Sox2* in EBs treated with or without ATV on days 1–6 and collected at the indicated times. Results are representative of >100 EBs/group. (**c**) Real-time PCR of *T* and *Sox2* in EBs treated with or without ATV and/or MVA during days 1–4 and collected on day 4. Results were analysed as in Fig. 1b. *P < 0.05, **P < 0.0001.

**Figure 3:**
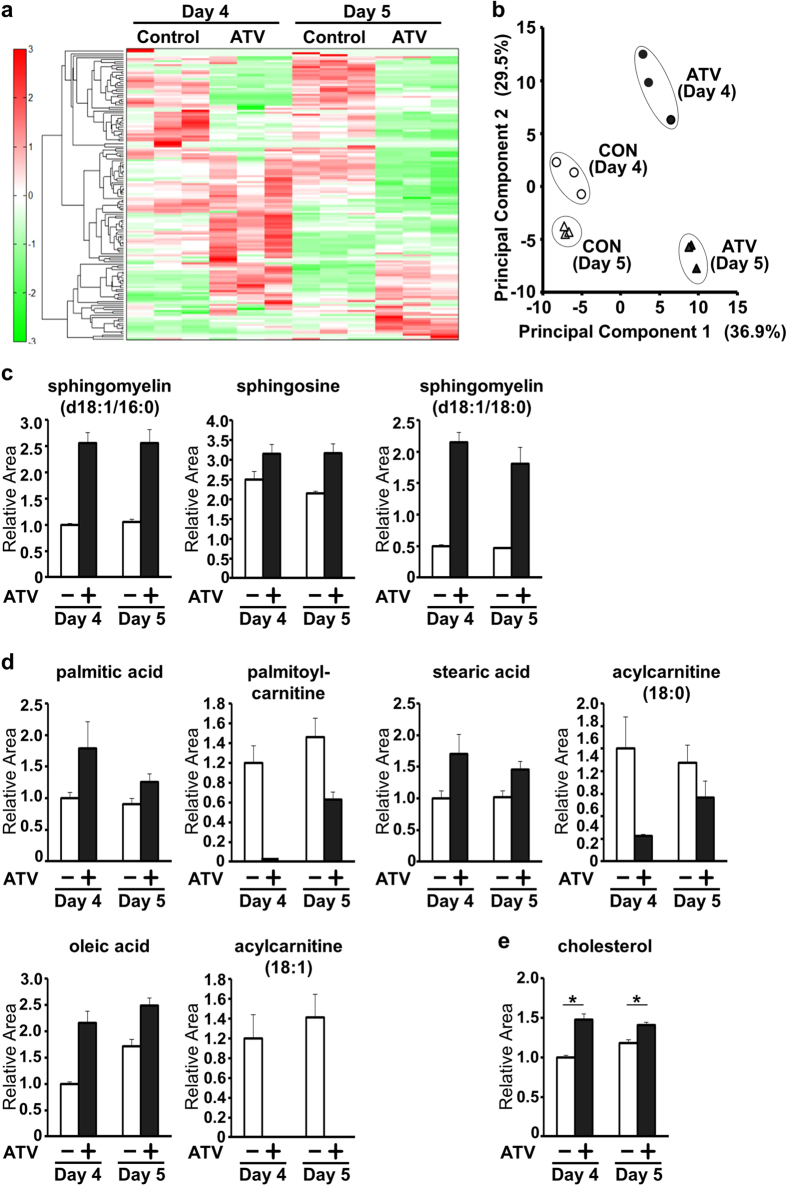
Metabolomic analysis of statin-treated mouse EBs. (**a**) Heat map showing differences in the profiles of 147 metabolites in EBs that were treated with or without ATV during days 3–5 and subjected to metabolomic analysis on days 4 and 5. (**b**) Principal component analysis of the EBs in (**a**). Principal components 1 and 2 account for 36.9% and 29.5% of total variance, respectively. (**c**) Intracellular levels of the indicated metabolites in the EBs in (**a**). Data are expressed as the relative area of signal peaks corresponding to each metabolite and represent the mean ± SD (n = 3). (**d**) Intracellular levels of the indicated free FAs and acylcarnitines in the EBs in (**a**) analysed as in (**c**). (**e**) Intracellular cholesterol levels in the EBs in (**a**) analysed as in (**c**). *P < 0.005.

**Figure 4:**
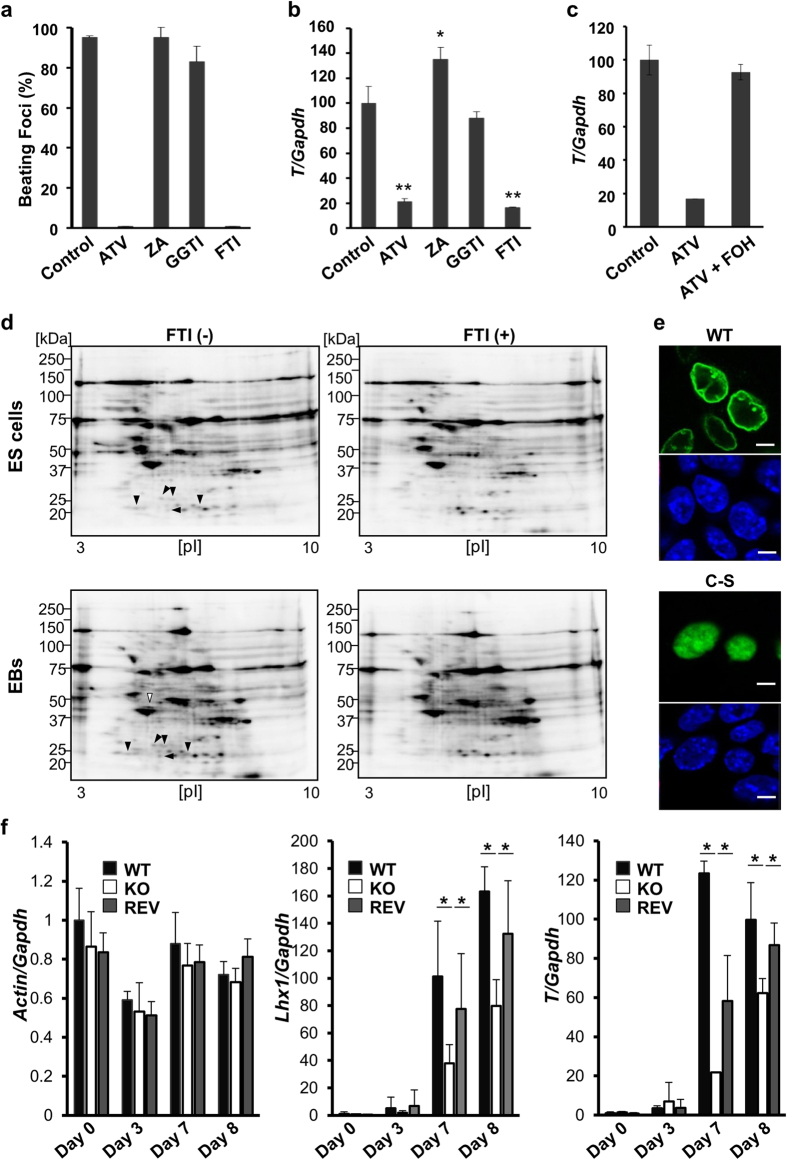
Identification of the effector pathway downstream of mevalonate. (**a**) Cardiomyocyte differentiation in EBs treated with DMSO (control) or 10 μM ATV, zaragozic acid (ZA), GGTI-2133 (GGTI) or FTI-277 (FTI) during days 3–6. Results were analysed as in Fig. 1c. (**b**) Real-time PCR of *T* expression in EBs treated with DMSO or 10 μM ATV, ZA, GGTI or FTI during days 3–4 and collected on day 4. Results were analysed as in Fig. 1b. *P < 0.05, **P < 0.001. *Zaragonic acid increased *T* and *Lhx1* expression, which might be due to upregulation of *HMGCR* levels on day 3 and 4 (Supplementary Figure 3e). (**c**) Real-time PCR of *T* levels in EBs treated with DMSO or 10 μM ATV, with/without 12.5 μM farnesol (FOH), during days 3–5 and collected on day 5. Results were analysed as in Fig. 1b. (**d**) Representative 2D-PAGE images of farnesylated proteins detected with biotin-phosphine from ES cells and EBs that were left untreated (left) or treated with 10 μM FTI-277 (right) and analysed on day 4. Results are representative of three cultures. Black arrowheads, ~20 kDa farnesylated proteins; white arrowhead, EB-specific farnesylated protein. (**e**) Immunostaining to detect the localisation of Flag-Myc-tagged Lamin B1 (WT) or Lamin B1 (C-S mutant) in ES cells. Nuclei were visualised by Hoechst 33342 staining. Scale bars, 5 μm. (**f**) Real-time PCR of the indicated genes in the Lamin B1 WT, KO and REV EBs. EBs were collected at the indicated times and analysed as in Fig. 1b. *P < 0.05.

**Figure 5:**
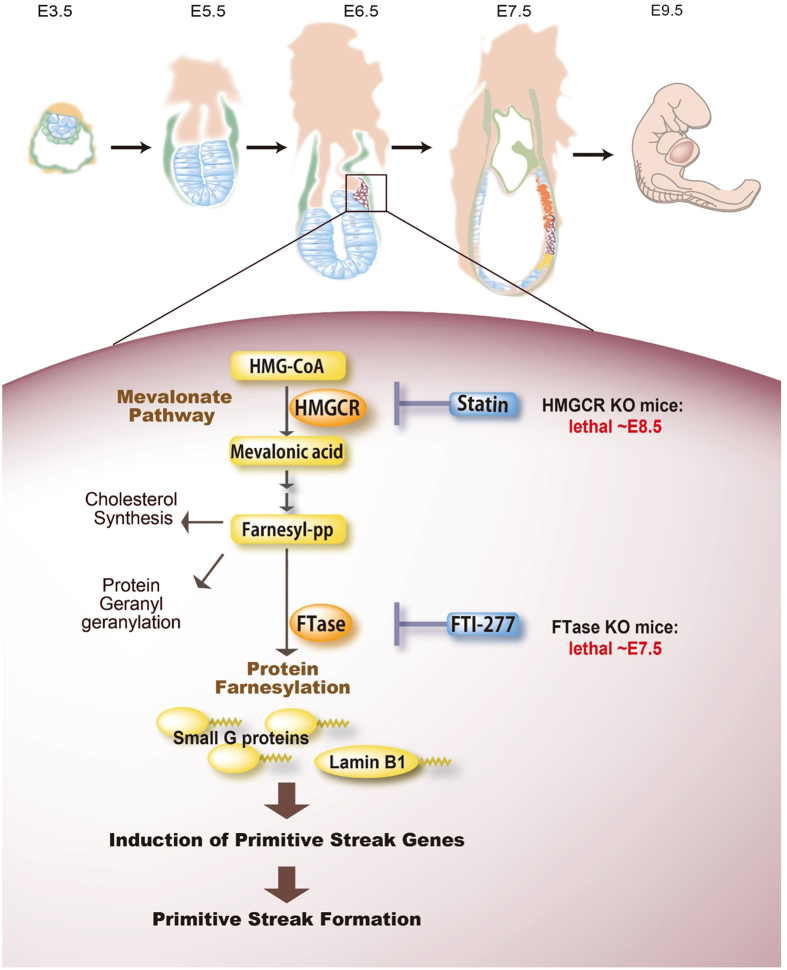
Involvement of the mevalonate pathway in primitive streak formation in mouse embryos. Top: Schematic diagram of mouse embryonic development on the indicated days. Bottom: The mevalonate pathway produces farnesyl diphosphate for cholesterol synthesis, protein geranylgeranylation and protein farnesylation. The farnesylation of various proteins, including small G proteins and the nuclear protein Lamin B1, triggers primitive streak formation. Statins inhibit the induction of primitive streak genes by suppressing protein farnesylation. Consistent with our observations, *Hmgcr* KO mice and FTase KO mice are embryonic lethal at E8.5 and E7.5, respectively^21^,^22^.
