# Supplementary material for: The mevalonate pathway regulates primitive streak formation via protein farnesylation
Source: Sci Rep. 2016 Nov 24;6:37697. doi: 10.1038/srep37697 (PMC5121603; doi:10.1038/srep37697)
Supplement: Supplementary Information [file srep37697-s1.pdf]

**Supplementary Information**

# The mevalonate pathway regulates primitive streak formation via protein farnesylation

Yoshimi Okamoto-Uchida[a,b], Ruoxing Yu[a], Norio Miyamura[a], Norie Arima[a], Mari Ishigami-Yuasa[c], Hiroyuki Kagechika[c,d], Suguru Yoshida[e], Takamitsu Hosoya[e], Makiko Nawa[f], Takeshi Kasama[g], Yoichi Asaoka[a], Reiner Wimmer Alois[h], Ulrich Elling[h], Josef M. Penninger[h], Sachiko Nishina[i], Noriyuki Azuma[i] and Hiroshi Nishina[a]

[a]Department of Developmental and Regenerative Biology, Medical Research Institute, Tokyo Medical and Dental University (TMDU), 1-5-45 Yushima, Bunkyo-ku, Tokyo, Japan

[b]Division of Medicinal Safety Science, National Institute of Health Sciences, 1-18-1 Kamiyoga, Setagaya-ku, Tokyo, Japan

[c]Chemical Biology Screening Center, Institute of Biomaterials and Bioengineering, TMDU, Tokyo, Japan

[d]Department of Organic and Medicinal Chemistry, Institute of Biomaterials and Bioengineering, TMDU, Tokyo, Japan

[e]Department of Chemical Bioscience, Institute of Biomaterials and Bioengineering, TMDU, Tokyo, Japan

[f]Laboratory of Cytometry and Proteome Research, TMDU, Tokyo, Japan

[g]Instrumental Analysis Research Division, Research Center for Medical and Dental Sciences, TMDU, Tokyo, Japan

[h]IMBA, Institute of Molecular Biotechnology of the Austrian Academy of Sciences, Vienna, Austria

[i]Department of Ophthalmology and Laboratory of Cell Biology, National Center for Child Health and Development, Tokyo, Japan.

**Supplementary Tables**

**Supplementary Table 1. Compounds identified as inhibiting primitive streak formation in mouse EBs.**

| Compound name | CAS No.* | Clinical and biological uses |
|---|---|---|
| Atorvastatin calcium | 134523-03-8 | Antihyperlipidemic,<br>HMG-CoA reductase inhibitor |
| Lovastatin | 75330-75-5 | |
| Cyclosporine | 59865-13-3 | Immunosuppressant,<br>Calmodulin inhibitor |
| Amitriptyline hydrochloride | 549-18-8 | Antidepressant,<br>5HT uptake inhibitor |
| Sertraline hydrochloride | 79559-97-0 | |
| Chlorprothixene | 113-59-7 | Antipsychotic |
| Norantiffein | 3691-03-0 | Psychostimulant |
| Aprofene | 3563-01-7 | Acetylcholine receptor inhibitor |
| Tretinoin<br>(all-trans retinoic acid) | 302-79-4 | Keratolytic |
| Honokiol | 35354-74-6 | Anti-tumorigenic activity, Anxiolytic<br>Anti-thrombotic |
| Propranolol | 525-66-6 | Antiarrhythmic |
| Papaverine hydrochloride | 61-25-6 | Smooth muscle relaxant |
| Helenin | 546-43-0 | Phytoncide; Anthelmintic |
| Chloramphenicol | 56-75-7 | Antibacterial, Antirickettsial |
| Bifonazole | 60628-96-8 | Antifungal, Calmodulin antagonist |

*CAS No., CAS Registry Number assigned by the Chemical Abstracts Service

**Supplementary Table 2. Gene ontology analysis of genes downregulated by statins.**

| Rank | Name | Score (p) |
|---|---|---|
| 1 | Anatomical structure morphogenesis (GO:0009653) | 1.54E-46 |
| 2 | Multicellular organismal process (GO:0032501) | 1.04E-43 |
| 3 | Cell differentiation (GO:0030154) | 2.02E-42 |
| 4 | Single-organism developmental process (GO:0044767) | 2.53E-41 |
| 5 | Anatomical structure formation involved in morphogenesis (GO:0048646) | 6.84E-41 |
| 6 | System development (GO:0048731) | 1.01E-40 |
| 7 | Single-multicellular organism process (GO:0044707) | 1.23E-40 |
| 8 | Developmental process (GO:0032502) | 1.99E-40 |
| 9 | Cellular developmental process (GO:0048869) | 3.07E-40 |
| 10 | Multicellular organismal development (GO:0007275) | 2.34E-39 |
| 11 | Anatomical structure development (GO:0048856) | 3.20E-39 |
| 12 | Regulation of multicellular organismal process (GO:0051239) | 5.28E-39 |
| 13 | Regulation of developmental process (GO:0050793) | 1.84E-35 |
| 14 | Organ development (GO:0048513) | 1.13E-34 |
| 15 | Tissue development (GO:0009888) | 2.59E-34 |
| 16 | Embryonic morphogenesis (GO:0048598) | 1.24E-33 |
| 17 | Positive regulation of biological process (GO:0048518) | 3.10E-33 |
| 18 | Response to external stimulus (GO:0009605) | 3.54E-31 |
| 19 | Gastrulation (GO:0007369) | 5.26E-31 |
| 20 | Embryo development (GO:0009790) | 6.36E-31 |

The top twenty GO categories for genes downregulated more than two-fold in ATV-treated EBs on days 3 and 4 as determined by microarray analysis.

**Supplementary Table 3. Metabolomic analysis of statin-treated EBs.**

Decreased metabolites

| Rank | Metabolite | m/z | Factor loading |
|---|---|---|---|
| 1 | Gly-Asp | 191.06 | −0.129 |
| 2 | Creatine | 132.08 | −0.127 |
| 3 | Thr | 120.07 | −0.125 |
| 4 | Butyrylcarnitine | 232.15 | −0.123 |
| 5 | Glycerophosphocholine | 258.11 | −0.123 |
| 6 | AC(14:0) | 372.31 | −0.122 |
| 7 | N-Acetylaspartic acid | 174.04 | −0.122 |
| 8 | O-Succinylhomoserine | 218.07 | −0.119 |
| 9 | Arg | 175.12 | −0.119 |
| 10 | ADP | 426.02 | −0.118 |

Increased metabolites

| Rank | Metabolite | m/z | Factor loading |
|---|---|---|---|
| 1 | Sphingomyelin(d18:1/16:0) | 703.58 | 0.129 |
| 2 | Sphingosine | 300.29 | 0.123 |
| 3 | Sphingomyelin(d18:1/18:0) | 731.61 | 0.122 |
| 4 | Cholesterol | 369.35 | 0.117 |
| 5 | 1-Oleoyl-glycero-3-phosphocholine | 522.36 | 0.116 |
| 6 | Oleic acid | 281.25 | 0.115 |
| 7 | Acetylcholine | 146.12 | 0.114 |
| 8 | Terephthalic acid | 165.02 | 0.113 |
| 9 | Stearic acid | 283.26 | 0.110 |
| 10 | 1-Hexadecyl-2-acetyl-glycero-3-phosphocholine | 524.37 | 0.108 |

Metabolites that were major contributors to principal component 1 are shown.

**Supplementary Table 4. Microarray analysis of statin-treated EBs.**

| Gene Symbol | Signal (Day3) | | Signal (Day4) | | Description |
|---|---|---|---|---|---|
| | Control | ATV | Control | ATV | |
| ***Fasn*** | **1094.5** | **2125.8** | **835.9** | **1575.9** | **fatty acid synthase** |
| ***Ldlr*** | **7901.7** | **15864.9** | **5132.4** | **11243.9** | **low density lipoprotein receptor** |

**Supplementary Table 5. Real-time PCR primers used in this study.**

| | | |
|---|---|---|
| *Gapdh* | F | GTCGGTGTGAACGGATTTGGC |
| | R | GTTGAGGTCAATGAAGGGGTCG |
| *Mhy7* | F | ACAACCCCTACGATTATGCGT |
| | R | ACGTCAAAGGCACTATCCGTG |
| *Map2* | F | GCCACAGTGGAGGAAGATTT |
| | R | ACCAGGCTTACTTGGCATCT |
| *Lhx1* | F | CCTCAACATGCGTGTTATCC |
| | R | CTGTTTCATCCTTCGCTCCT |
| *Wnt3* | F | CACACACGAGGACGGAGAA |
| | R | CCAGTCGCACAATCTACCCC |
| *Brachyury T* | F | CCCATTTGCTAAAGCCTTCCT |
| | R | GTCCCCCGGTTCCTCCATTA |
| *Sox2* | F | CCCACCTACAGCATGTCCTA |
| | R | GTGGGAGGAAGAGGTAACCA |
| *Sox17* | F | CAGTAAGCCAGATTTGGTCTCTGA |
| | R | CCAAGACCTCTTGGGGAAATAGG |
| *Gata6* | F | CAAAAGCTTGCTCCGGTAAC |
| | R | TGAGGTGGTCGCTTGTGTAG |
| *Wnt8* | F | TTTGGAGAAAGGGAAGGATG |
| | R | CATGACACTTGCAGGTCCTT |
| *Gsc* | F | AGCCAAGTGGAGACGACAGAA |
| | R | TTGAGGACGTCTTGTTCCACTTC |
| *Fgf10* | F | AGGGGAAACTCTATGGCTCA |
| | R | ATTTGCCTGCCATTGTGCTG |
| *Actin* | F | GGACATCCGCAA AGACCTGTA |
| | R | GCTCAGGAGGAGCAATGATCT |
| *Sox1* | F | GCAGCGTTTCCGTGACTTTAT |
| | R | GGCAGAACCACAGGAAAGAAA |
| *Otx2* | F | GACGTTCTGGAAGCTCTGTT |
| | R | ATGGTTGGGACTGAGGTACT |
| *zcMhc2* | F | TTGGCTGTGAGATCTGCATT |
| | R | CAGAGGAACGATGTGAGCAG |
| *zNestin* | F | AGGATCAAGAGGCAGATGCT |
| | R | ATCAGGCTGTCAATCTGCTG |

F, Forward primer; R, Reverse primer; z, zebrafish.

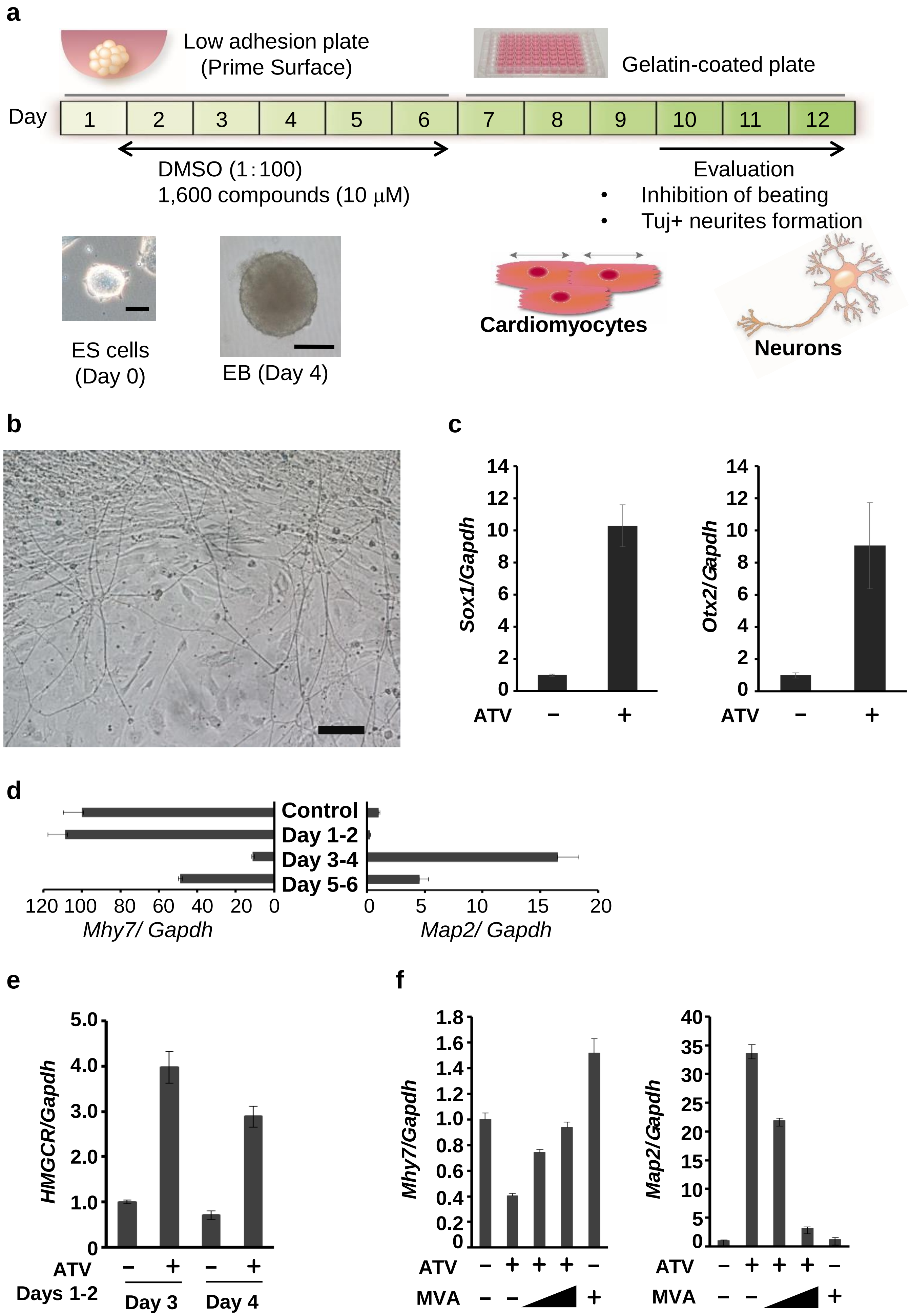

a
Low adhesion plate
(Prime Surface)
Gelatin-coated plate
Day
1
2
3
4
5
6
7
8
9
10
11
12
DMSO (1:100)
1,600 compounds (10 μM)
Evaluation
• Inhibition of beating
• Tuj+ neurites formation
ES cells
(Day 0)
EB (Day 4)
Cardiomyocytes
Neurons
b
c
Sox1/Gapdh
Otx2/Gapdh
ATV
–
+
d
Control
Day 1-2
Day 3-4
Day 5-6
Mhy7/ Gapdh
Map2/ Gapdh
e
HMGCR/Gapdh
ATV
Days 1-2
Day 3
Day 4
f
Mhy7/Gapdh
Map2/Gapdh
ATV
MVA

**Supplementary Figure 1. Statins inhibit cardiomyogenesis and induce neurogenesis in mouse EBs.** (**a**) High-throughput screening of 1,600 well-known drugs using mouse EBs. Top: Screening workflow. EBs were cultured in suspension in low cell adhesion 96-well plates for 6 days. Each compound (10 μM) was added to the culture medium during days 2–6. EBs were transferred to gelatin-coated 96-well plates on day 6, and their differentiation was determined during days 10–12 by evaluating the inhibition of cardiac beating and the presence of β-tubulin III (Tuj)-positive neurites. Lower left: Light microscopy of ES cells on day 0 and an EB on day 4. Scale bars, 20 μm and 100 μm, respectively. (**b**) Microscopic image of neurites in an EB that was treated with 10 μM ATV during days 1–6 and evaluated on day 12. Scale bar, 250 μm. (**c**) Real-time PCR analysis of *Sox1* and *Otx2* in EBs treated with or without 10 μM ATV during days 3-6. EBs were collected on day 10. (**d**) Real-time PCR analysis of the cardiomyocyte marker *Mhy7* and the neural marker *Map2* on day 10 in EBs treated with DMSO (control) or 10 μM ATV for the indicated periods. (**e**) Real-time PCR of *HMGCR* expression in control or ATV-treated EBs during days 1-2. EBs were collected on day 3 and 4. (**f**) Real-time PCR of *Mhy7* and *Map2* expression in the EBs in Figure 1e, collected on day 12. mRNA levels were normalised to *Gapdh* expression. Results represent the mean ± SD (n = 3) from three experiments.

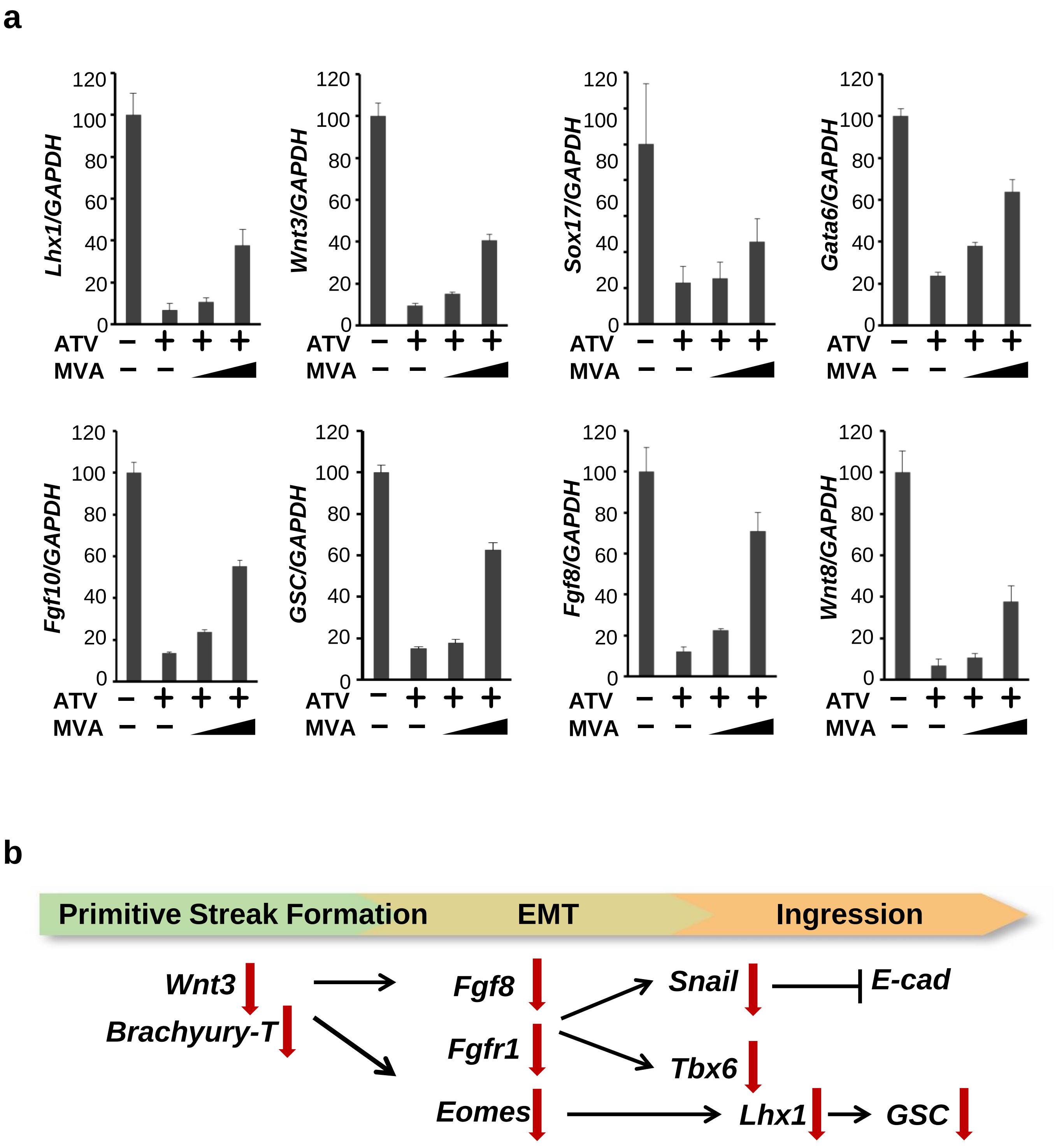

a
Lhx1/GAPDH
Wnt3/GAPDH
Sox17/GAPDH
Gata6/GAPDH
Fgf10/GAPDH
GSC/GAPDH
Fgf8/GAPDH
Wnt8/GAPDH
ATV
MVA
b
Primitive Streak Formation
EMT
Ingression
Wnt3
Brachyury-T
Fgf8
Fgfr1
Eomes
Snail
E-cad
Tbx6
Lhx1
GSC

**c**

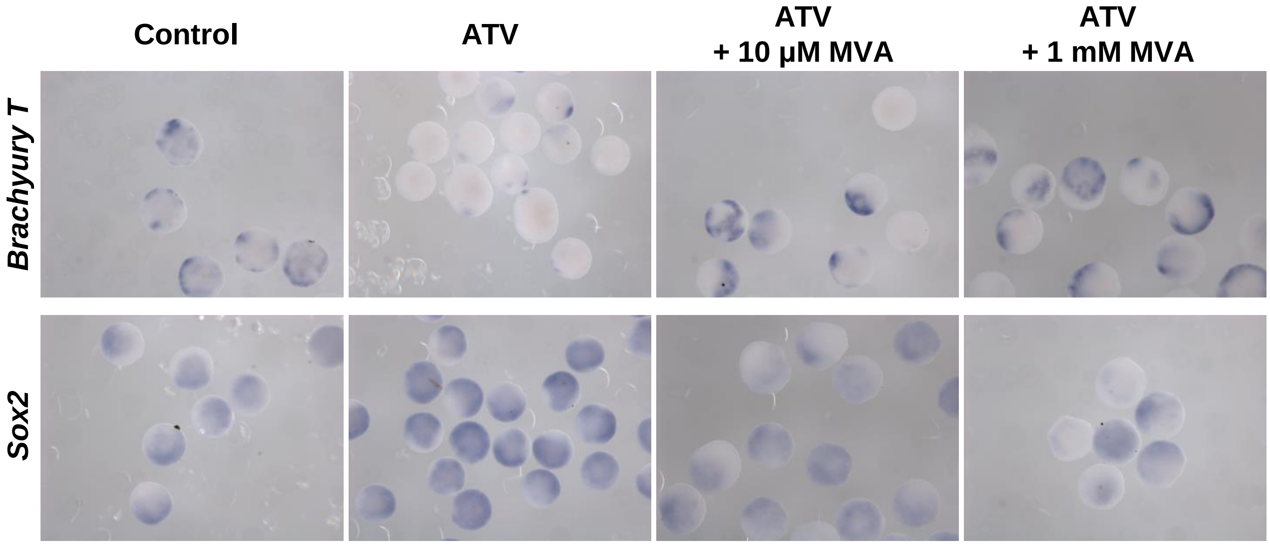

Control
ATV
ATV
+ 10 μM MVA
ATV
+ 1 mM MVA
*Brachyury T*
*Sox2*


**d**

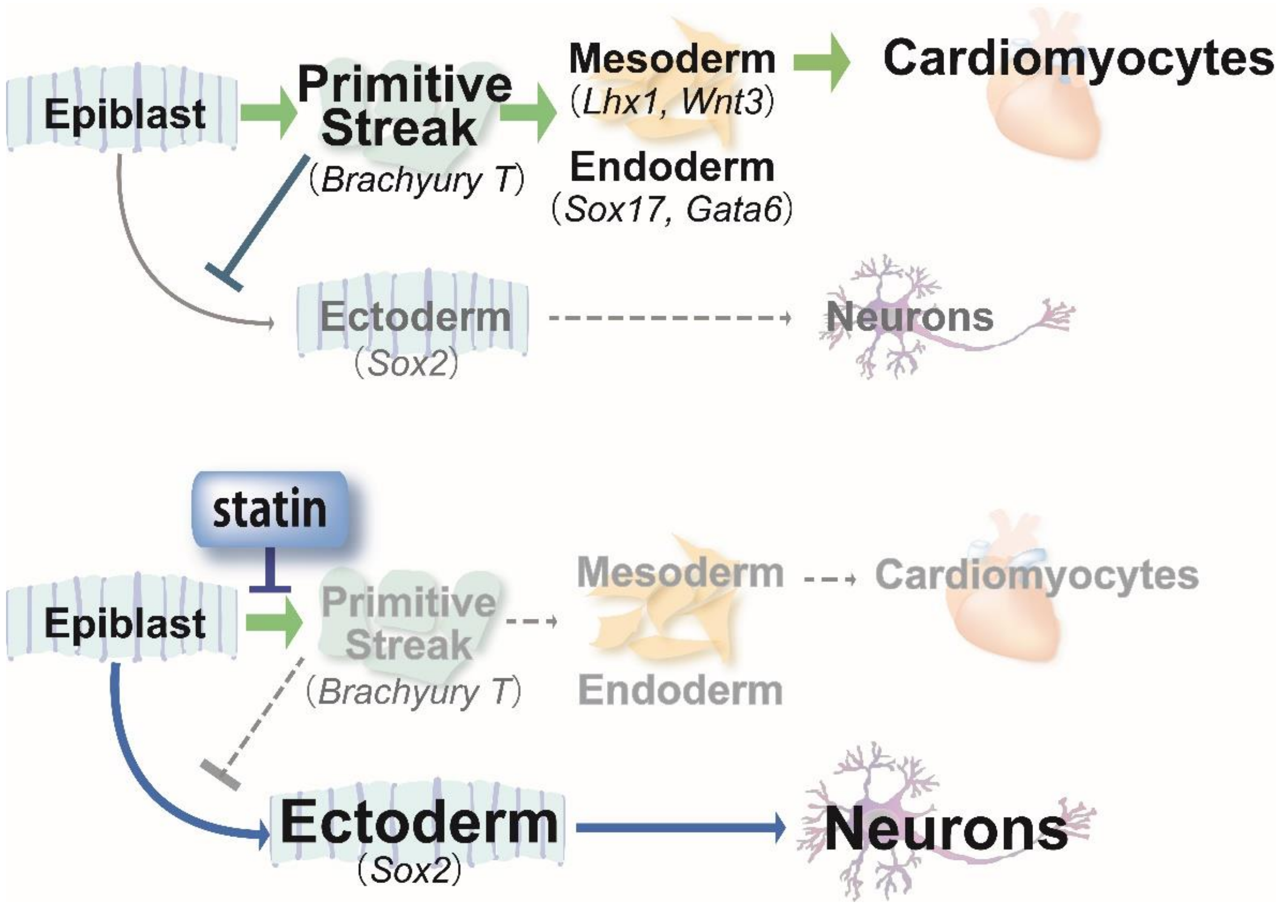

Epiblast
Primitive
Streak
(*Brachyury T*)
Mesoderm
(*Lhx1*, *Wnt3*)
Endoderm
(*Sox17*, *Gata6*)
Cardiomyocytes
Ectoderm
(*Sox2*)
Neurons
statin
Epiblast
Primitive
Streak
(*Brachyury T*)
Mesoderm
Endoderm
Cardiomyocytes
Ectoderm
(*Sox2*)
Neurons

**Supplementary Figure 2. The effects of statins on the differentiation of the three germ layers.**

(**a**) Real-time PCR of *Lhx1, Wnt3, Sox17, Gata6, Fgf10, GSC, Fgf8* and *Wnt8* in EBs treated with or without ATV and/or MVA during days 1–4 and collected on day 4. Results were analysed as described in Supplementary Figure 1c. (**b**) Schematic diagram of the transcriptional cascade during mouse embryo gastrulation, which is triggered by primitive streak formation. Representative genes that regulate each step are indicated. Genes that were downregulated in EBs by ATV treatment are indicated with red arrows. (**c**) *In situ* hybridisation to detect *T* and *Sox2* in control, 10 μM ATV treated or ATV plus 100 μM or 1 mM MVA treated EBs. EBs were treated with each drug during days 1-4 and collected at day 5. Results are representative of >100 EBs/group. (**d**) A schematic model of how statins may alter ES cell fate. The primitive streak is formed from epiblast cells and induces the differentiation of mesoderm and endoderm, and cardiomyogenesis. This process inhibits neuroectodermal differentiation. When primitive streak formation is blocked by statin treatment, epiblast cells commit to the neuroectodermal lineage, reducing cardiomyocyte differentiation.

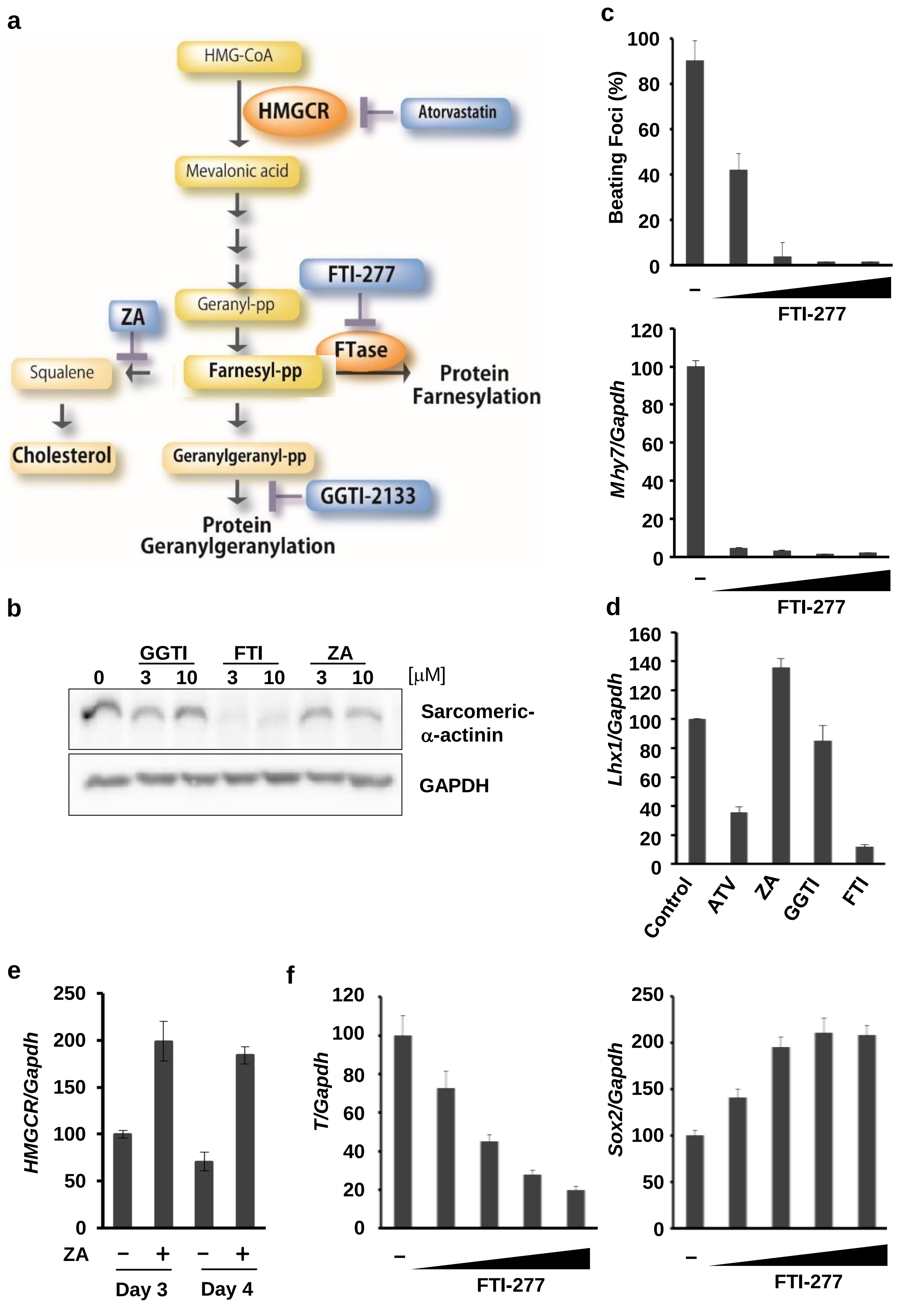

a
HMG-CoA
HMGCR
Atorvastatin
Mevalonic acid
FTI-277
Geranyl-pp
ZA
FTase
Squalene
Farnesyl-pp
Protein Farnesylation
Cholesterol
Geranylgeranyl-pp
GGTI-2133
Protein Geranylgeranylation
b
GGTI
FTI
ZA
0
3
10
3
10
3
10
[μM]
Sarcomeric-α-actinin
GAPDH
c
Beating Foci (%)
FTI-277
Mhy7/Gapdh
FTI-277
d
Lhx1/Gapdh
Control
ATV
ZA
GGTI
FTI
e
HMGCR/Gapdh
ZA
Day 3
Day 4
f
T/Gapdh
FTI-277
Sox2/Gapdh
FTI-277

**Supplementary Figure 3. The effect of farnesyltransferase inhibitor on mouse EB differentiation.**

(**a**) Schematic overview of the mevalonate pathway involving cholesterol synthesis, protein geranylgeranylation and protein farnesylation. Metabolites (yellow), enzymes (orange) and chemical inhibitors (blue) are shown. (**b**) Western blotting to detect sarcomeric α-actinin in EBs treated as indicated during days 3–6 and assessed on day 10. Results were analysed as in Figure 1d. (**c**) EBs were treated with 1, 2, 4 or 8 μM FTI-277 during days 3–6 and cardiomyocyte differentiation (upper) was analysed as in Figure 1c, or *Mhy7* expression (lower) was analysed as in Supplementary Figure 1c. (**d**) Real-time PCR analysis of *Lhx1* in EBs that were treated with DMSO (control) or 10 μM ATV, ZA, GGTI or FTI during days 3–4 and collected on day 4. (**e**) Real-time PCR of *HMGCR* expression in control or ZA-treated EBs during days 3-4. EBs were treated with 10 μM ZA during days 3-4 and collected at the end of day 3 and on day 4. (**f**) Real-time PCR analysis of *T* and *Sox2* in EBs that were treated with 1, 2, 4 or 8 μM FTI-277 during days 3–4 and collected on day 4.

**a**

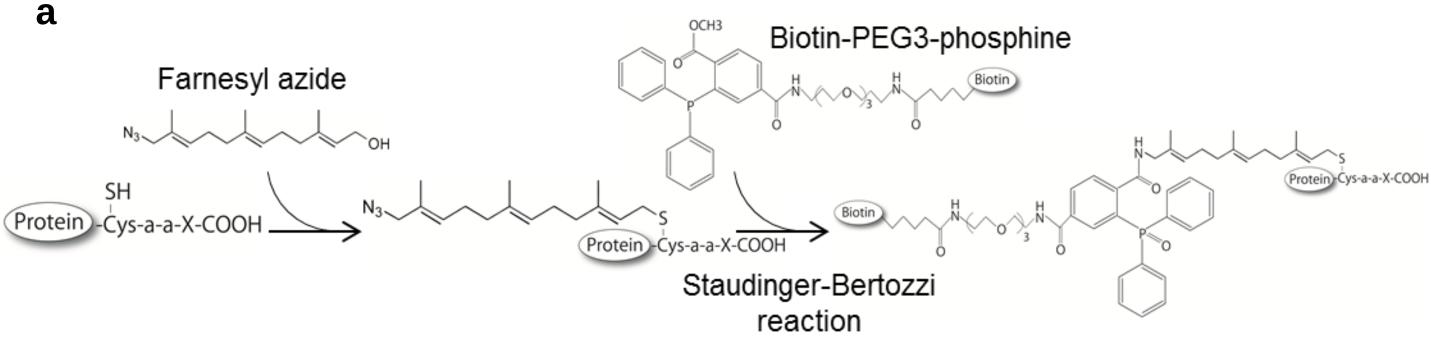


**b**

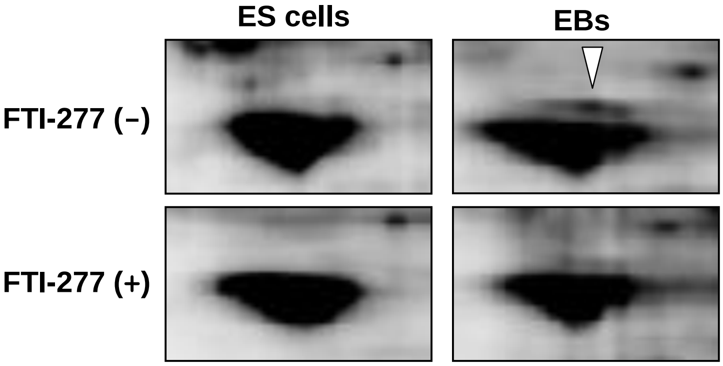


**c**

LaminB1
MATATPVQQQRAGSRASAPATPLSPTRLSRLQEKEELRELNDRLAVYIDKVRSLETENSALQLQVTEREEVRGR
ELTGLKALYETELADARRALDDTARERAKLQIELGKFKAEHDQLLLNYAKKESDLSGAQIKLREYEAALNSKDAA
LATALGDKKSLEGDLEDLKDQIAQLEASLSAAKKQLADETLLKVDLENRCQSLTEDLEFRKNMYEEEINETRRKH
ETRLVEVDSGR**QIEYEYK**LAQALHEMREQHDAQVR**LYKEELEQTYHAK**LENAR**LSSEMNTSTVNSAR**EELME
SRMRIESLSSQLSNLQKESRACLERIQELEDMLAKERDNSRRMLSDREREMAEIRDQMQQQLSDYEQLLDVKL
ALDMEISAYR**KLLEGEEERLKLSPSPSSRVTVSRASSSR**SVRTTRGKRKRVDVEESEASSSVSISHSASATGNV
CIEEIDVDGKFIRLKNTSEQDQPMGGWEMIR**KIGDTSVSYKYTSR**YVLKAGQTVTVWAANAGVTASPPTDLIWK
**NQNSWGTGEDVKVILKNSQGEEVAQR**STVFKTTIPEEEEEEEEEPIGVAVEEER**FHQQGAPR**ASNKS**C**AIM

**Supplementary Figure 4. Identification of farnesylated proteins in mouse EBs.**

(**a**) Schematic diagram outlining the TAS approach using biotin-phosphine. Farnesyl azide, a bioorthogonal farnesol analogue, is incorporated into substrate proteins by endogenous farnesyltransferase (FTase) in cells. Azide-labelled proteins were covalently bound to biotin-phosphine by the Staudinger-Bertozzi reaction *in vitro*. (**b**) Magnified TAS blots described in Figure 4d are shown. EB-specific 42 kDa spot is indicated with white arrowhead. (**c**) The amino acid sequence of mouse Lamin B1. Peptide fragments identified by MS/MS analysis are underlined. The farnesylation site is boxed.

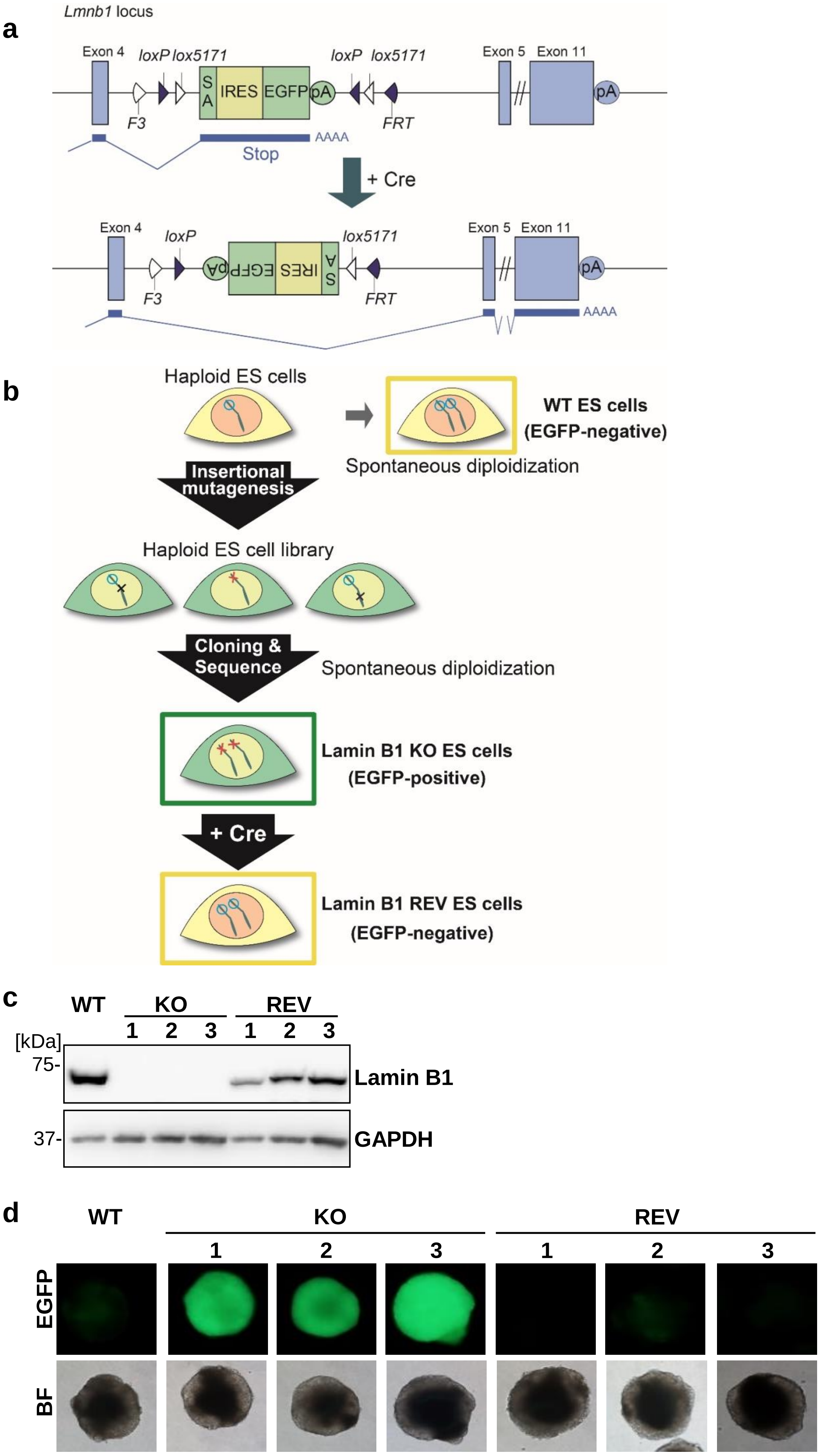

a
Lmnb1 locus
Exon 4
loxP
lox5171
SA
IRES
EGFP
pA
loxP
lox5171
F3
FRT
Exon 5
Exon 11
pA
AAAA
Stop
+ Cre
Exon 4
loxP
lox5171
F3
FRT
Exon 5
Exon 11
pA
AAAA
b
Haploid ES cells
WT ES cells
(EGFP-negative)
Spontaneous diploidization
Insertional mutagenesis
Haploid ES cell library
Cloning & Sequence
Spontaneous diploidization
Lamin B1 KO ES cells
(EGFP-positive)
+ Cre
Lamin B1 REV ES cells
(EGFP-negative)
c
WT
KO
REV
1 2 3 1 2 3
[kDa]
75-
37-
Lamin B1
GAPDH
d
WT
KO
REV
1 2 3 1 2 3
EGFP
BF

**Supplementary Figure 5. Preparation of Lamin B1-deficient and –revertant ES cells.**

(**a**) Top: The targeting vector inserted into the *Lmnb1* locus encoding Lamin B1. The gene terminator cassette blocks Lamin B1 mRNA synthesis. White and black arrowheads represent the Cre recombinase target sites *loxP*, *lox5171*, *FRT* and *F3* [27]. Blue rectangles are the fourth, fifth and eleventh exons of the *Lmnb1* gene. Thick and thin blue lines represent exonic and intronic portions of pre-mRNAs, respectively. SA, splice acceptor sequence; pA, poly(A) addition signal. Bottom: Cre recombinase inverts the gene terminator cassette, restoring *Lmnb1* gene expression. (**b**) Schematic diagram of Lamin B1 generation in KO ES cells (EGFP-positive) using haploid genetics. Lamin B1 REV ES cells (EGFP-negative) were generated from KO ES cells by transient Cre expression. (**c**) Western blotting to detect Lamin B1 in WT ES cells, three Lamin B1 KO clones and three Lamin B1 REV clones. Results were analysed as in Figure 1d. (**d**) Representative fluorescent (EGFP) and bright field images of EBs from WT, KO and REV ES cells.

**a**

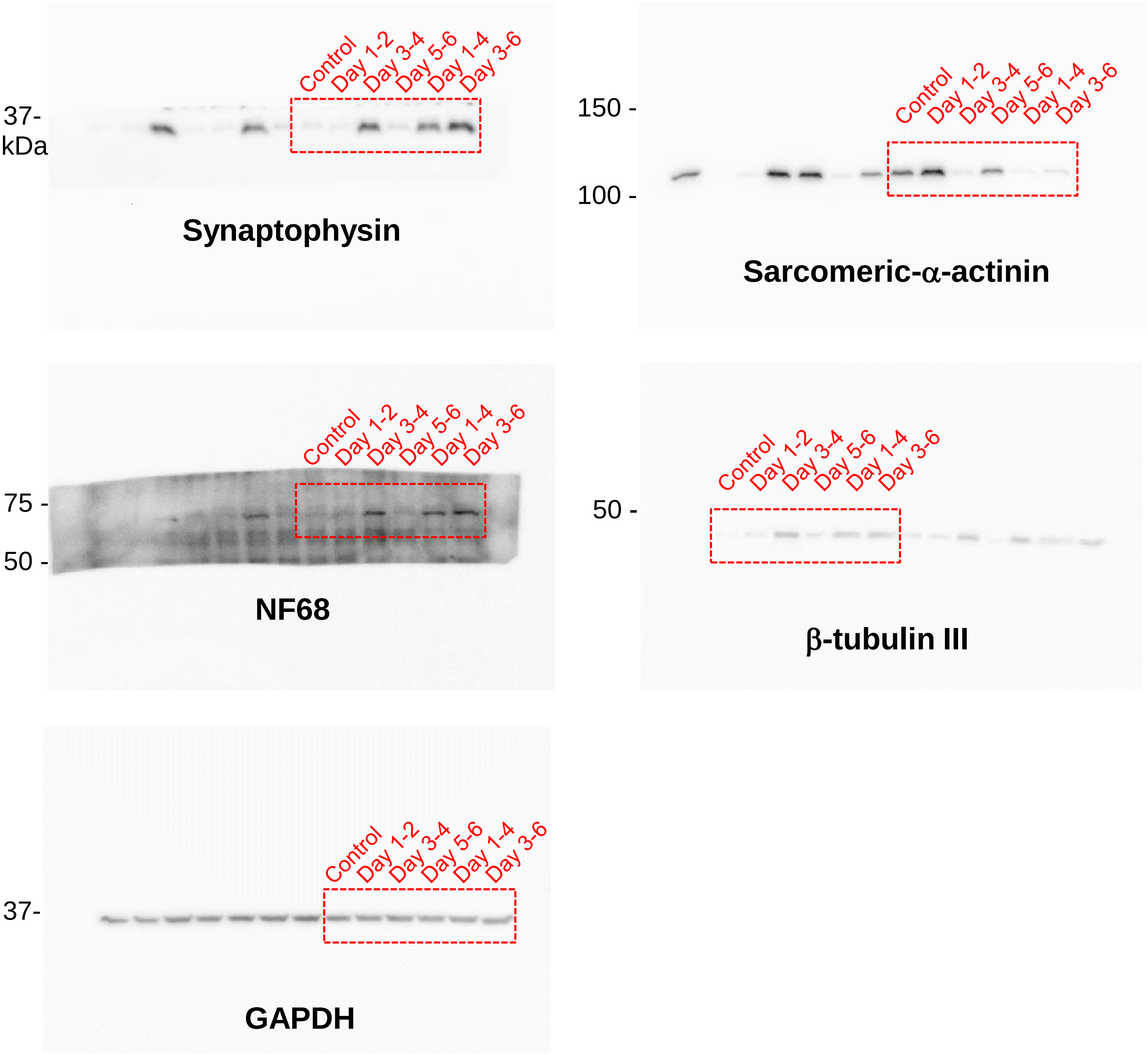
Control
Day 1-2
Day 3-4
Day 5-6
Day 1-4
Day 3-6
37-
kDa
Synaptophysin
150 -
100 -
Sarcomeric-α-actinin
75 -
50 -
NF68
50 -
β-tubulin III
37-
GAPDH

**b**

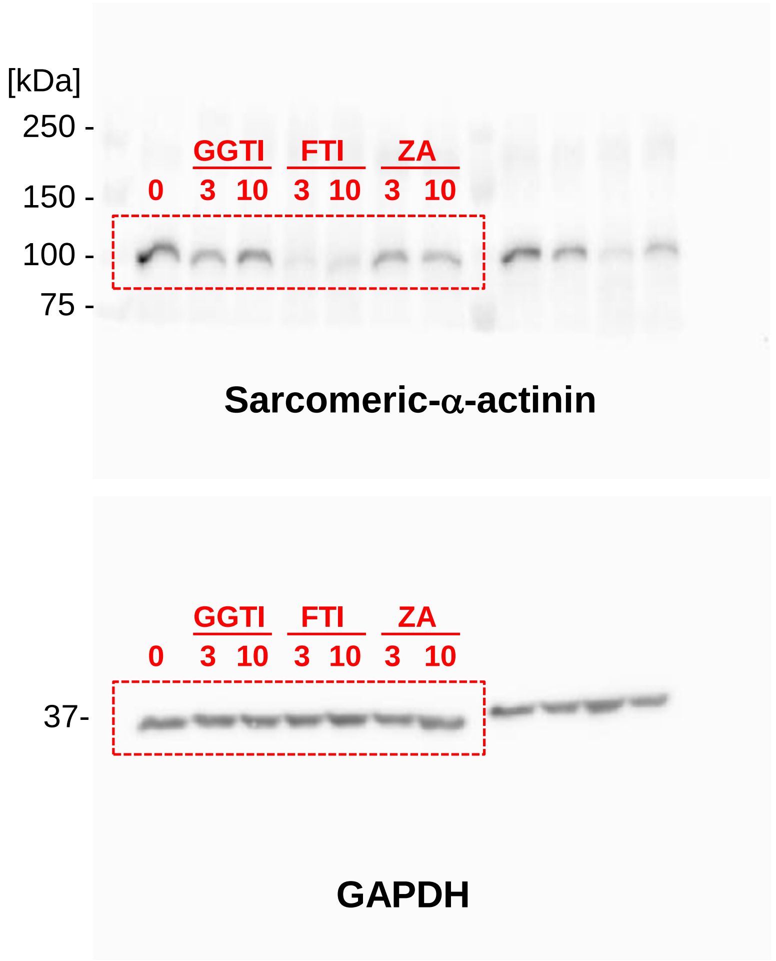
[kDa]
250 -
150 -
100 -
75 -
GGTI
FTI
ZA
0
3
10
3
10
3
10
Sarcomeric-α-actinin
GGTI
FTI
ZA
0
3
10
3
10
3
10
37-
GAPDH

**c**

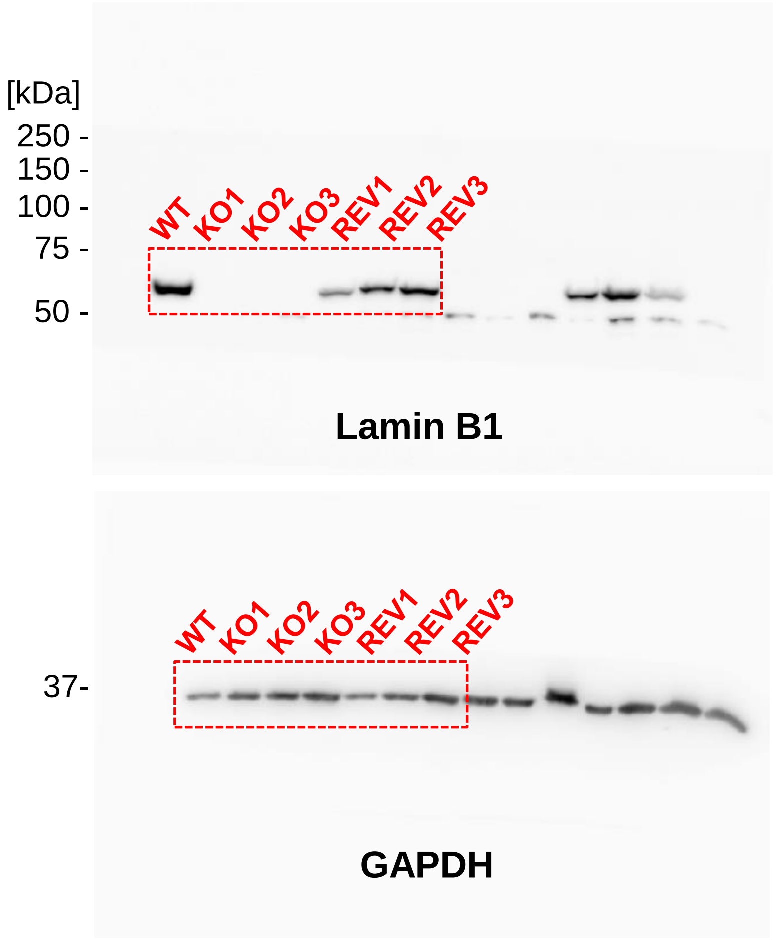
[kDa]
250 -
150 -
100 -
75 -
50 -
WT
KO1
KO2
KO3
REV1
REV2
REV3
Lamin B1
WT
KO1
KO2
KO3
REV1
REV2
REV3
37-
GAPDH

**Supplementary Figure 6. Uncropped Western Blot images.** Full blots for Western blotting data of Figure 1d (a), Supplementary Figure 3b (b) and Supplementary Figure 5c (c) are shown. Red boxes highlight the cropped segment presented in main or supplementary figures.
